# Optimizing Laboratory Rearing of a Key Pollinator, *Bombus impatiens*

**DOI:** 10.3390/insects12080673

**Published:** 2021-07-26

**Authors:** Erin Treanore, Katherine Barie, Nathan Derstine, Kaitlin Gadebusch, Margarita Orlova, Monique Porter, Frederick Purnell, Etya Amsalem

**Affiliations:** Department of Entomology, Center for Chemical Ecology, Center for Pollinator Research, Huck Institutes of the Life Sciences, Pennsylvania State University, University Park, PA 16802, USA; kib5501@psu.edu (K.B.); ntd34@psu.edu (N.D.); kmg6027@psu.edu (K.G.); mfo5180@psu.edu (M.O.); msp36@psu.edu (M.P.); fsp5027@psu.edu (F.P.); eua6@psu.edu (E.A.)

**Keywords:** bumble bees, mating, CO_2_ narcosis, social insects, social cues, solitary phase, reproduction

## Abstract

**Simple Summary:**

Rearing insects in captivity is critical for both research and commercial purposes. One group of insects that stands out for their ecological and scientific importance are bumble bees. However, most research studies with bumble bees rely on commercial colonies due to the challenges associated with initiating colonies in the laboratory. This limits the ability to study the early stages of the colony life cycle and the ability to control for variables such as colony age and relatedness. To overcome these challenges, we tested several aspects related to the solitary phase of the North American bumble bee species, *Bombus impatiens*. In this species, queens emerge by the end of the season, mate, and enter a winter diapause. They then initiate a colony the next spring. We examined the optimal age for mating and how the timing of CO_2_ narcosis (a technique used to bypass diapause) and social cues post-mating affect queen egg laying. We demonstrate an optimum age for mating in males and females and the importance of worker and pupa presence to egg laying in queens. Overall, our findings indicate that the laboratory rearing of bumble bees can be improved by reducing challenges associated with rearing queens during the solitary phase.

**Abstract:**

Bumble bees are key pollinators for wild and managed plants and serve as a model system in various research fields, largely due to their commercial availability. Despite their extensive use, laboratory rearing of bumble bees is often challenging, particularly during the solitary phase queens undergo before founding a colony. Using a literature survey, we demonstrate that most studies rely on commercially available species that are provided during the colony’s social phase, limiting study on early phases of the life cycle and the ability to control for colony age and relatedness. Laboratory rearing is challenging since the queen solitary phase is less understood compared to the social phase. To overcome this barrier, we examined several aspects related to the queen solitary phase: the effect of age on likelihood of mating, how the timing of CO_2_ narcosis post-mating (a technique to bypass diapause) affects egg-laying, and whether different social cues affect the success of colony initiation. Our data show an optimum age for mating in both sexuals and decreased egg-laying latency in the presence of workers and pupae. The timing of CO_2_ narcosis did not significantly affect egg laying in queens. These findings can be incorporated to improve bumble bee rearing for research purposes.

## 1. Introduction

Bumble bees are critical pollinators in natural ecosystems, greenhouses, and field-based agriculture [1,2]. They also serve as an important model species for research in various fields such as social behavior [3], insect cognition [4], sexual selection [5], and chemical ecology [6]. Among the approximately 250 *Bombus* species around the world, a subset has been cultivated to provide pollination services. *Bombus terrestris* Linnaeus (Hymenoptera: Apidae) (Asia and Europe) and *Bombus impatiens* Cresson (Hymenoptera: Apidae) (North America, Canada, and Mexico) comprise the majority of cultivated colonies worldwide, estimated in 2006 at one million colonies annually [1]. The commercial availability of these species makes them a suitable model species to work with, as evidenced by their extensive use in research. However, there are limits to the information researchers can gain when working with colonies that were primarily optimized to meet commercial pollination needs. Moreover, rearing practices used by commercial companies are often proprietary and unpublished, thus limiting the ability of researchers to successfully rear colonies independently.

Most of the bumble bee research in North America is conducted using the widespread and abundant species *B. impatiens*. Colonies of this species are annual and founded by a single queen following a period of winter diapause [7] or after a CO_2_ narcosis that causes queens to bypass diapause and initiate a colony [8]. Often, commercial colonies are lacking information on the duration of diapause and whether queens were kept in cold storage or treated with CO_2_, both of which have been shown to impact colony life cycle trajectories, such as the number and timing to producing sexuals [9,10]. Moreover, information on mating, inbreeding, or genetic relatedness between colonies is often not available. Prior to entering diapause, queens mate and are assumed to avoid mating with related males [11]. However, in captivity, it has been shown that queens in several species (*B. impatiens, B. hypnorum*, *B. bifarius*, *B californicus*, *B. frigidus*, *B. huntii*, and *B. rufocinctus*) can mate more than once [5,12,13], and inbreeding in a number of bumble bee species (*B. terrestris, B. rufocinctus, B. californicus,* and *B. impatiens*) may occur frequently [13,14,15]. Finally, due to constraints associated with shipping and because commercial colonies are primarily used to provide pollination services, colonies are typically shipped to research laboratories with a few dozen workers, about 3–5 weeks after the emergence of the first worker. This substantially limits the amount of information that can be gained on the early phases of the life cycle. Approximately 4–5 weeks after the emergence of the first worker, colonies transition into a competition phase where the queen is no longer the sole producer of eggs [15,16]. Therefore, older colonies are impractical for studying reproductive conflicts at the colony level [3]. Additionally, workers sampled from colonies for use in queenless cages may retain physiological characteristics of their natal colony age [17,18,19], posing a significant challenge to behavioral and physiological studies that are conducted using commercial colonies. Despite the widespread use of *B. impatiens*, there remains a dearth of published data available on mating behavior and successful colony initiation. The ability to rear colonies in the lab can provide researchers with more control over mating, diapause regime, genetic relatedness, and colony age, but it is less common due to the obvious challenges associated with initiating colonies in the laboratory.

Successful in-laboratory rearing of colonies from single queens requires understanding and replicating the natural conditions during mating, diapause, colony initiation, and colony maintenance during the social phase. The former requires minimal effort. However, the solitary phase is much less understood and harder to replicate in the laboratory. Most annual bumble bee species inhabit temperate and arctic regions and produce only one generation per year [3,20,21]. New queens emerge toward the end of the colony life cycle, leave their natal colony, and mate with conspecific males before they enter a winter diapause of approximately 6–9 months [22]. The following spring, queens emerge from their hibernaculum, search for a suitable nest site, and forage for nectar and pollen [7]. Ovary activation and egg laying will follow, resulting in the emergence of workers and the beginning of the social phase. Mating in the lab can be challenging and requires a specific set of environmental and social conditions, and previous studies showed significant variation in mating success (25–68%) [23,24]. Following mating, queens are placed in diapause-like conditions (cold storage) for a period of several months to stimulate reproduction; however, maintaining queens in a months-long cold storage significantly slows down the rearing process and can result in high levels of mortality (>90%) [25]. A more efficient method for laboratory rearing of *B. terrestris* has been developed by Roseler (1985), whereby cold storage is replaced with CO_2_ narcosis that induces a rapid transition to reproduction. While CO_2_ narcosis is widely used in both bumble bee research and commercial rearing, numerous factors related to its application are not well understood, for example, the optimal timing of narcosis following mating, the duration of CO_2_ exposure, and the most effective number of exposures. Finally, initiating a colony following diapause or CO_2_ narcosis requires a social stimulus that can be challenging to both researchers rearing colonies from newly emerged queens or from spring wild-caught queens. Previous studies have used workers, brood, and wax to initiate egg laying in newly mated queens, and a study in three North American bumble bees showed that without a social cue, less than 25% of the queens produced workers successfully [26,27]. However, the nature of the social cue in different species is still unclear.

Another challenge of laboratory rearing stems from the diversity across bumble species in their behavior, biology, and life history strategies. Even the most frequently used species for research, *B. terrestris* and *B. impatiens,* exhibit fundamental differences in their rates of worker reproduction [15,28,29], aggressive behavior [15,30], and the number of times a queen is mated [15], thereby preventing the generalization of findings from one species to another and emphasizing the necessity for standardized protocols. Moreover, a review of mating behaviors across bumble bee species found significant variation in the number of preferred mates, mating duration, use of sperm plugs, and pre-mating behavior [31]. Queens kept in cold storage have been also shown to differ in their preferred environmental conditions during diapause. For example, *B. hypocrita* queens kept in cold storage (5 °C, 4 months) preferred dry vermiculite in place of soil, while *B. ignitus* queens preferred moist (50–55% moisture) vermiculite or peat moss [32]. CO_2_ narcosis has been shown to induce a transition to reproduction in *B. impatiens*, *B. hypnorum*, *B. pratorum*, *B. terrestris*, and *B. vestalis* [8,33,34,35]. Thus, its mode of action may be similar across species, but this has been poorly studied in non-commercially available species. Lastly, some variation was also demonstrated in the social cue necessary for inducing egg laying in different species. In *B. terrestris*, egg laying was stimulated by adding older larvae or young pupae [34,36,37], another queen [38], workers [39], wax, or honey bee workers [40]. Inducing egg laying by housing queens with honey bee workers or another queen was also demonstrated in *B. appositus*, *B. bifarius*, and *B. centralis* [26]. However, studies in *B. humilis*, *B. ruderarius*, and *B. hortorum* showed that the pairing of queens reduced rates of egg laying [41,42], and the addition of honey bee workers was unsuccessful in *B. impatiens* [15], stressing the need for a more nuanced approach in the laboratory rearing of individual bumble bee species.

In this study, we conducted a literature review to examine how bumble bee species are being sourced for research and examined factors affecting rearing success during the solitary phase in *B. impatiens*. This species is both an economically and ecologically important pollinator and the most commonly used *Bombus* species for research in North America. Specifically, we examine the effects of age on the likelihood of mating in both queens and males, how the timing of CO_2_ narcosis post-mating affects the initiation of egg laying, and how different social cues affect egg laying during the colony initiation phase. We hypothesize that in *B. impatiens*, mating success is age dependent in both sexes, that delayed CO_2_ narcosis will result in delayed egg laying, and that colony foundation will be faster when queens are provided with workers and/or pupa. We discuss how our findings can be used to optimize rearing of *B. impatiens* colonies in the laboratory for research purposes.

## 2. Methods

### 2.1. Literature Review

We performed a systematic literature review using the most recent 150 published studies that were conducted using bumble bees to examine which and how bumble bee species were used and where the colonies were sourced from. The literature review was performed on 15 September 2020 using the Web of Science. The search terms ((Bombus*) OR (Bumble bees*) OR (Bumblebees*)) yielded a total of 6220 results. We read the title and abstract of the articles to remove review papers, studies that exclusively surveyed wild populations, or those that did not use live bumble bees. The abstract and methods of papers that met these criteria were read in full to gather information about (A) where the bees were sourced from, (B) what caste or life stages were used, and (C) which species were used. We used the following categories to describe the source of bees: (1) commercial colonies, (2) lab-reared colonies that were initiated in the lab either using reproductives that were caught in the wild or using reproductives from commercial colonies; (3) field-collected bees that were brought to lab for the study but not reared in the laboratory. If the study used multiple sources of bees, such as commercial colonies and field-collected samples, we only listed the main substantially used group. We further describe the bumble bee species that was used. Species that were used in less than 1% of the studies were listed under “other”. We further listed the component of the colony used in the study: queens, workers, males, brood, or a combination of the above.

### 2.2. Queen and Male Collection

Reproductive producing colonies of *B. impatiens* were obtained from Koppert Biological Systems (Howell Michigan, USA) and maintained in the laboratory under darkness, a temperature of 28–30 °C, 60% relative humidity, and supplied ad libitum with 60% sucrose solution and honey bee collected pollen (light spring bee pollen, purchased from Swarmbustin’ Honey). Colony maintenance and sampling of individuals were performed under red light.

Queens were collected upon eclosion and placed in small cages (11 cm diameter × 7 cm height) containing ad libitum 60% sucrose solution and light spring honey bee-collected pollen (Swarmbustin’ honey). Fresh pollen was provided every other day. Separation from parental colonies (where males may be present) is necessary to prevent inbreeding, which can negatively affect colony size and the number of sexuals [43,44]. Queens and males were then tagged with paint on their thoraxes using non-toxic bee marking pens (Uni-Posca). Males were collected upon eclosion from their natal colonies and placed into small cages with other males eclosing on the same day from the same natal colony. Males were kept under the same conditions as queens. Natal colony identity was recorded in all experiments as it may affect bee physiology and behavior [45,46].

### 2.3. Experimental Setup

#### 2.3.1. Effect of Queen and Male Age on Mating

To examine the effect of queen age on mating success, 50 queens underwent daily mating trials between August and November of 2019. Queens were placed in a mating arena with unrelated males that were at least three days old. Mating conditions are described below. The first trial was conducted one day after queen eclosion. Trials were repeated daily until the queens mated or reached the age of 14 days, whichever came first. To examine the effect of male age on mating success, 99 males underwent daily mating trials between February and July of 2020. Males were placed in a mating arena with unrelated queens (*n* = 76) that were between the ages of 3 and 14 days of age. The first trial was conducted when the males were 3 days old and was repeated daily until the male had mated or reached the age of 16 days, whichever came first.

#### 2.3.2. Effect of CO_2_ Narcosis Timing on Colony Initiation

To examine the effect of CO_2_ narcosis timing on colony initiation, we used 26 queens from the experiment examining the impact of queen’s age on mating. Following mating, the queens underwent a CO_2_ narcosis (see below) at the following time points: within 6 h of mating (*n* = 9), 48 ± 6 h after mating (*n* = 9), and 72 ± 6 (*n* = 8) hours after mating. The queens were then placed in individual cages supplied with pollen and nectar and with two newly emerged workers that were replaced every 3 days to prevent worker–queen aggression (Roseler 1985). Cages were checked daily for egg laying for a period of 28 days, and the latency to the first egg-laying event was recorded.

#### 2.3.3. Effect of Social Cues on Colony Initiation

Mated queens from the experiment examining the impact of male’s age on mating (*n* = 44) were used to examine the effects of different social cues on colony initiation in queens. Following mating, queens were transferred back into their individual cages and treated with CO_2_ within a period of 24 h. Queens were then randomly assigned to 1 of 4 treatment groups according to the social cue provided to them: (1) no social cue (*n* = 14), (2) 3–5 fresh pupae that were replaced every 3 days (*n* = 10), (3) 2 newly emerged workers that were replaced every 3 days (*n* = 9), or (4) 3–5 fresh pupae and 2 newly emerged workers that were replaced every 3 days (*n* = 11). Old pupae were left in the cages if egg cups were built on top of the pupal case. Cages were checked daily for egg laying during a period of 28 days, and the latency to the first egg laying event was recorded.

### 2.4. Mating Conditions

Queens and males were placed in a plastic flying arena (35 × 20 × 12 cm) with wire mesh and/or holes drilled on the sides for ventilation. The mating arena was lined with a paper towel. Each mating arena contained 2–5 queens and at least 2–3 unrelated males for every queen. Mating chambers were supplied with fresh pollen and 60% sucrose solution at all times. Mating chambers were placed in ambient light and room temperature for 2–4.5 h per day and observed approximately every 15 min. In the remainder of the time, queens were kept in individual cages to prevent mating. Any pairs observed mating were removed from the mating chamber and placed back into an individual cage until the mating ended. Mating pairs remain connected for at least 30 min.

### 2.5. CO_2_ Narcosis

CO_2_ narcosis of queens was performed using the protocol described by Amsalem and Grozinger 2017. A steady stream of pure CO_2_ was applied to a tape-sealed cage containing the queen through a single hole for one minute. Under these conditions, CO_2_ reaches nearly 100% within seconds and remains at this level for the remainder of the one-minute period. Queens were observed to lose mobility within approximately 20 s and were kept in the cage and placed back into the temperature-controlled chamber for an additional 30 min before the tape was removed. During these 30 min, CO_2_ concentration in the cage decreases rapidly until ambient environmental levels are achieved.

### 2.6. Sampling of Newly Emerged Workers and Pupae

Newly emerged workers and pupae were used to examine the social cues needed for colony initiation. Workers were collected from queen-right colonies less than 24 h after eclosion and kept with queens in pairs. Workers were replaced every 3 days to prevent aggression toward the queens. Newly emerged workers are easily distinguishable by their silvery appearance. Brood cells of pupae were carefully removed from the natal colony using forceps and used only if they remained intact during collection. Groups of 4–6 pupae were placed into queen cages and were replaced every 3 days unless they had egg cups or cells built on top of them.

### 2.7. Statistical Analyses

Statistical analyses were performed using SPSS v.21. (SPSS, Chicago, IL), and data visualizations were performed using RStudio (Version 3.5.3, RStudio: Integrated Development for R, Boston, MA, U.S.A.). The effect of age on mating was analyzed using chi-square analyses with Monte Carlo permutation tests (10,000 random permutations) to evaluate whether the observed frequencies of mating at each age were different from predicted random mating. The effects of the timing of CO_2_ narcosis and of social cues on egg-laying latency in queens were examined using a generalized linear mixed models analysis (hence, GLMM). Latency to egg-laying data were analyzed using a Poisson distribution with log link and latency as the dependent variable, CO_2_ narcosis/social cue treatment as a categorical predictor, and parental colony as a random factor. Because queens varied in their age at mating, we also examined for a relationship between queen age and latency to egg-laying in both the CO_2_ and social cue experiments using a simple linear regression. Post hoc pairwise comparisons were performed using the least significant difference (LSD) method for the social cue data. Robust estimation was used to handle violations of model assumptions. Best-fitting models were selected based on the AICc value or QICc, and statistical significance in all analyses was accepted at *p* < 0.05.

## 3. Results

### 3.1. Literature Review

**Figure 1:**
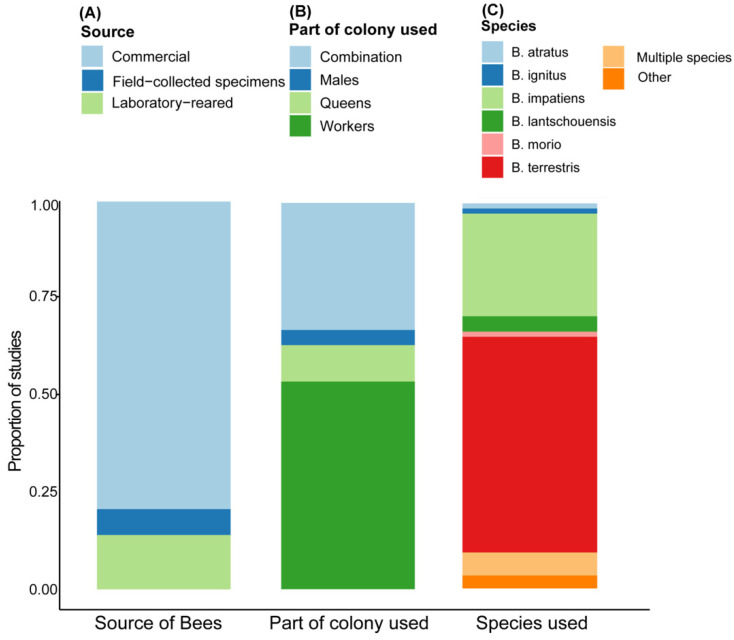
A literature review of the common uses of bumble bees in research studies. We surveyed the most recent 150 published papers with bumble bees as a study system to examine three aspects: (**A**) whether bumble bees were sourced commercially, lab reared, or field collected in the study; (**B**) which caste (queens, workers, males) was used or if multiple castes/life stages were used in combination; and (**C**) which species were used in the study. For more information, please refer to the text.

The literature review showed that most studies rely on commercially available colonies (79.3%, *n* = 119/150, Figure 1A) followed by the use of laboratory-reared colonies (14%, *n* = 21/150). A little more than half of these studies were conducted using workers (54.6%, *n* = 82/150, Figure 1B) followed by the use of a combination of castes and life stages (32%, *n* = 48/150). Lastly, a vast majority of these studies (82.6%) were conducted using either *B. impatiens* (26.6%, *n* = 40/150, Figure 1C) or *B. terrestris* (56%, *n* = 84/150). The third mostly commonly used species was *B. lantschouensis*, which was used in 4% of studies.

### 3.2. The Effect of Queen and Male Age on Mating

**Figure 2:**
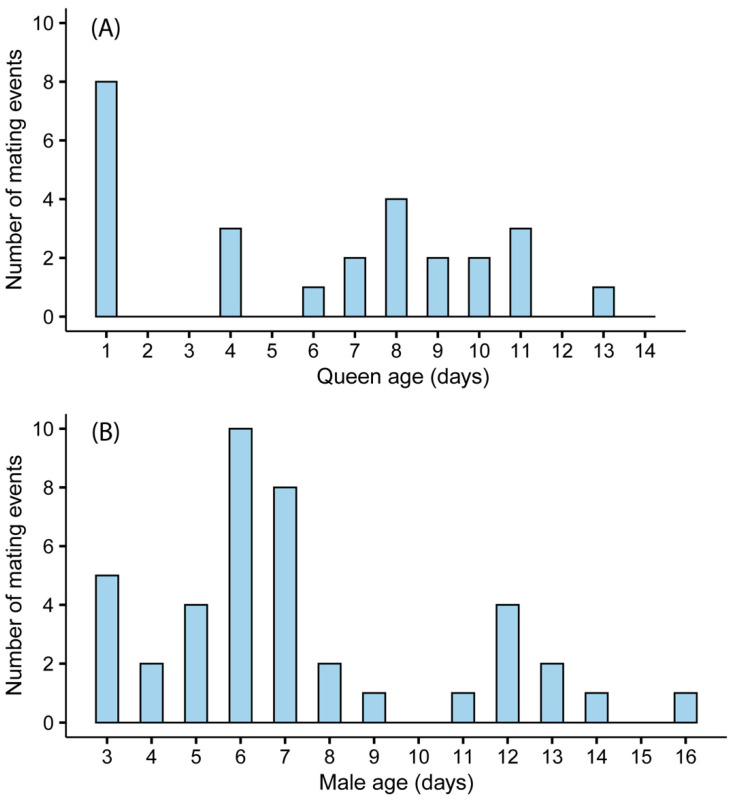
The impact of age on mating in *Bombus impatiens* queens (**A**) and males (**B**). Forty-nine queens were given the opportunity to mate daily between the ages of 1 and 14 days. Of these, 26 were successfully mated. Ninety-nine males were given the opportunity to mate daily between the ages of 3 and 16 days. Of these, 41 were successfully mated.

In total, 49 of the 50 queens were given the opportunity to mate everyday (one died prematurely), and 53% (*n* = 26) of them were observed mating over the 14-day period. The age of queens had a significant effect on the likelihood of mating (*n* = 478 mating opportunities, χ^2^_13_ = 26.15, *p* = 0.015; Figure 2A); however, the likelihood of mating was also affected by parental colony (χ^2^_2_ = 12.12, *p* = 0.002). Therefore, we also performed a generalized estimating equations model (hence, GEE) with age as our predictor variable and parental colony as a subject variable to control for interdependencies in the data and found that age still significantly affected mating (GEE; Wald χ_2_^2^ = 13.30, *p* = 0.001; Figure 2A). Our results showed that 54% (*n* = 14) of the mating occurred at the age of 6–11 days, and there was a 16% (*n* = 8/49) chance a queen would mate at 1-day-old if given the opportunity. In the parallel experiment, 41% (*n* = 41) of the males mated over a period of 14 days. The age of males had a significant effect on the likelihood of mating, with 76% (*n* = 31) of the mating events occurring at the age of 3–8 days (*n* = 1033 mating opportunities, χ^2^_13_ = 31.13, *p* = 0.003; Figure 2B); the likelihood of mating was not affected by the parental colony (χ^2^_7_ = 6.55, *p* = 0.482; Figure 2B).

### 3.3. The Effect of CO_2_ Narcosis Timing on Colony Initiation

**Figure 3:**
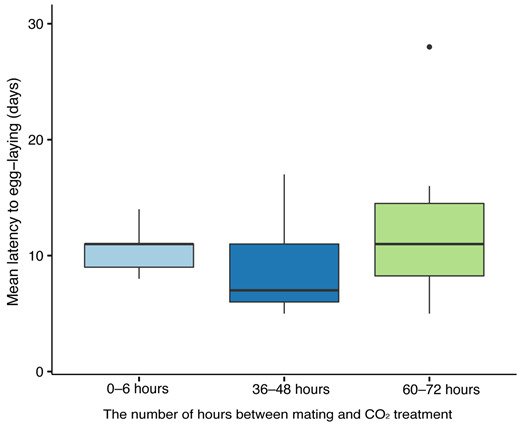
The impact of CO_2_ treatment on the latency to lay eggs in *Bombus impatiens* queens. Queens (*n* = 26) were treated with CO_2_ at three time points after mating: 0–6 h (*n* = 9); 36–48 h (*n* = 9); 60–72 h (*n* = 8). Queens were monitored for egg laying for 28 days. Boxplots represent the minimum, lower quartile, median (bolded line), upper quartile, and maximum values; points represent outliers.

The timing of CO_2_ narcosis did not significantly affect the latency to egg laying in queens (GLMM: (F_2,23_ = 0.97, *p* = 0.40, Figure 3). Parental colony was also not significant as a random effect (*p* = 0.31), and there was no correlation between queen age at mating and latency to egg laying (r = 0.082, *p* = 0.69, *n* = 26). The median age when queens laid eggs was 11 days when CO_2_ was administrated 6 h after mating, 7 days when CO_2_ was administered 36–48 h after mating, and 11 days when CO_2_ was administered 60–72 h after mating. In all three treatment groups, 100% of queens laid eggs.

### 3.4. The Effect of Social Cues on Colony Initiation

**Figure 4:**
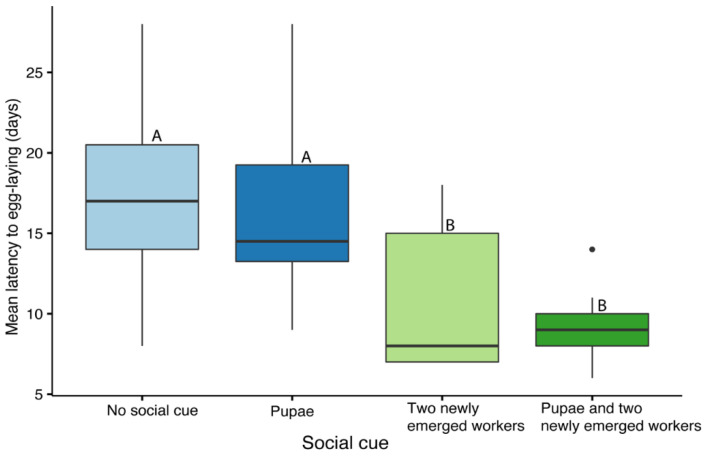
The effect of social cues on colony initiation in *Bombus impatiens*. Queens (*n* = 39/44) were treated with CO_2_ and exposed to four different social cues: no social cue (*n* = 14), 3–5 fresh pupae (*n* = 10), 2 newly emerged workers (*n* = 9), and 2 newly emerged workers and 3–5 fresh pupae (*n* = 11). Queens were monitored for egg laying for 28 days. Boxplots represent the minimum, lower quartile, median (bolded line), upper quartile, and maximum values; points represent outliers. Different letters indicate statistical differences at α = 0.05.

The social cue significantly affected latency to egg laying (GLMM: F_3,35_ = 8.428, *p* = 0.001, Figure 4). The effect of the parental colony on egg laying was not significant (*p* = 0.35), and there was no correlation between queen age at mating and latency to egg laying (r = 0.164, *p* = 0.32, *n* = 39). Post hoc pairwise comparisons showed that queens kept with two newly emerged workers with or without pupae laid eggs significantly sooner compared to queens that were kept with no social cue or with only pupae. All queens excluding one in the ‘no cue’ treatment laid eggs. Queens that did not lay eggs during the experimental period of 28 days were terminated and included as right-censored data. Mortality was not substantial (5 out of 44 queens) but varied across the treatments with zero mortality in queens housed with pupae or newly emerged workers, three queens housed with no social cue, and two queens housed with pupae and workers. In all cases, the reason for mortality was unknown.

## 4. Discussion

In this study, we use a literature review to examine how bumble bees are being used in research studies and demonstrate that a majority of the research is performed using commercial colonies and commercially available species. The most dominant species used was *B. terrestris* followed by *B. impatiens*. More than 75% of these studies were sourcing colonies for the exclusive use of workers or multiple castes and life stages. Still, the information researchers can gain by using commercial colonies in their studies is limited due to shipping constraints and limited information about colony age and genetics. The alternative is to initiate colonies in the laboratory from reproductives caught in the wild or reared in commercial colonies. However, this option is also challenging since standardized methods for rearing queens during the solitary phase are often unpublished. To remove some of these barriers, we conducted a series of experiments to examine queen preferences during mating, diapause (or lack of it), and colony initiation. We focus on the most commonly used species in North America, *B. impatiens*, and demonstrate that the laboratory rearing of *B. impatiens* can be improved by expanding our knowledge about the preferences of sexuals during the solitary phase. We identified an optimum age for mating in queens and males, both were more likely to mate at the ages of 6–11 and 3–8 days, respectively. We also found that all CO_2_-treated queens laid eggs regardless of the time we administrated CO_2_ narcosis, confirming previous findings on the effectiveness of CO_2_ narcosis for bypassing diapause in captivity [34,39]. Finally, we demonstrated that providing queens with newly emerged workers, either with or without pupae, reduced the time until egg laying was initiated when compared to queens maintained alone or with pupae.

Mating in both *B. impatiens* queens and males was affected by age. However, there were differences in the preferred age for mating between sexes. The impact of age is in line with previous findings showing that male sexual maturity, queen physiological quality, and mating behaviors in both sexes change with age [22,24,45,47,48]. Previous studies in *B. terrestris* showed that 6–7-day-old queens were the most receptive to mating and that a majority of mating events occur before queens were 11 days old, in line with the optimum age range found in the current study (6–11 days) [24,48,49]. Small portions of our queens were observed to mate at the age of 1–2 days old, similar to findings reported in *B. terrestris* queens [24]. This is likely an artifact of laboratory rearing (where queens and males are not allowed the leave the colony) and may be attributed to the inability of queens to resist male mating attempts. Early adult life (days 1–5) in queens was repeatedly shown to be a critical period for acquiring nutrients during both diapause and colony foundation [12,22,45], and, while the outcomes of early mating are unknown, it may negatively affect food consumption [50]. This underscores the need to separate queens from males upon eclosion to prevent premature mating and to delay mating until the optimal age range for each sex. Such a strategy will not only increase mating success but also the success of the resulting colonies since early mating with siblings may result in inbreeding and may also impair the development of the resulting colonies [14,51]. Our data also show that most males (71%) mated before the age of 10 days and males were most likely to mate between the ages of 3 and 8 days. This is slightly earlier compared to the findings reported for *B. terrestris* males, where the average age at mating was 12.1 ± 1.3 days [24]. Here, too, some males younger than 5 days were observed to mate, but, again, this may be unique to laboratory rearing since male sexual maturity is achieved at later ages [24,52].

Age is not the only factor affecting mating success. Mating is a complicated process that depends upon multiple variables. Previous studies have stressed the importance of light conditions [53], temperature [48], male mass and length [47], and colony population source [54]. In this study, all mating experiments were conducted at room temperature (~21 °C), under natural light, with all bees kept in total darkness when not in the mating chambers. Similar conditions were tested in *B. terrestris*, where no significant differences in mating were observed in ambient temperatures of 17–26 °C [48]. Furthermore, all of the colonies that were used in our study were sourced from the same regional supplier, reducing the likelihood of any differences related to the population source. We kept a constant ratio of males to queens, which has also been shown to influence mating behavior [47]. Controlling for these multiple variables is critical for maintaining a replicable rate of mating in the above age range.

The 100% success in egg laying following CO_2_ narcosis confirms previous studies and justifies the use of this technique to bypass diapause over other alternatives [8,34]. However, the time between mating and CO_2_ narcosis does not seem to matter or to affect egg-laying success in *B. impatiens*. This is in line with previous work in *B. terrestris* showing successful initiation of a colony following CO_2_ treatment, despite modifications in the number of CO_2_ applications [34], the duration of CO_2_ exposure, and the timing to administrate the CO_2_ treatment (i.e., 5 vs. 20–30 d after mating) [33,39], suggesting that there is a fair amount of flexibility in the way CO_2_ is applied. More importantly, these results emphasize that queens can achieve high rates of survival and egg laying following CO_2_ narcosis, both of which can be drawbacks of using cold storage [55,56]. With that being said, CO_2_ narcosis can affect queen behavior, physiology, and the development of resulting colonies; however, the impacts are not always negative. For example, *B. impatiens* queens exposed to CO_2_ narcosis increased their flight activity and aggression levels, and their immune functions were improved when faced with a bacterial challenge [8]. Additionally, colonies headed by queens treated with CO_2_ were shown to produce significantly more workers, males, and queens than colonies headed by queens that were kept in cold storage [9]. Therefore, while using CO_2_ narcosis is an effective technique for bypassing diapause in laboratory settings, it presents numerous tradeoffs, each of which should be weighed according to individual research needs.

The final stage of the solitary phase, the initiation of a colony, is challenging for both queens reared in the laboratory and wild-caught springtime queens. While some queens will lay eggs without any additional cues, the overall success can be improved by providing a social cue. The speed is important not only for the sake of saving time and increasing efficiency but also for the overall size and productivity of the resulting colonies [40]. However, this step is highly variable across species that may differ in the cues they require. Our data show that the overall percentage of queens laying eggs within 3 weeks in all treatment groups was very high (95%). However, eggs were laid faster in the presence of newly emerged workers with or without pupae compared to either pupae alone or the absence of pupae and workers. For comparison, in *B. hypocrita*, using non-conspecific newly emerged workers resulted in twice as many workers produced in the first month of colony initiation, and the use of pupae resulted in a 40% increase in the percentage of queens laying eggs compared to the absence of a social cue [57,58]. Additionally, in *B. terrestris*, the presence of young male pupae, their age, and orientation in the cage were shown to decrease the time until egg laying [37]. Overall, our findings suggest that providing the queens with a few newly emerged workers can accelerate the initiation of a new colony.

Bumble bees are an excellent model organism to study various research questions. Relying solely on commercial colonies limits the amount of information researchers can extract and reduces their ability to control for confounding factors that have been shown to affect bee behavior and physiology. The ability to initiate colonies from mated queens in the lab allows for much more flexibility in experimental design and for new questions to be asked. Our data suggest a few practical ways to minimize the barriers in rearing queens and colonies in captivity: (1) separate queens from their natal colonies upon eclosion and provide them with unlimited access to sucrose solution and fresh pollen; (2) control for queen and male age at mating to increase mating success; (3) control for mating conditions, including light, temperature, and size of mating arena; (4) apply CO_2_ narcosis following mating—this can be carried out immediately or several days after mating; and (5) provide the queens with several newly emerged workers to accelerate egg laying and replace them every few days to prevent queen–worker aggression. Following these guidelines resulted in 50% mating success and nearly 100% colony initiation success.

Although our study focused on *B. impatiens*, our results may be useful for improving laboratory rearing of other bumble bee species. For example, despite differences in mating strategies across species, there are similarities in the preferred age of mating between *B. terrestris* and *B. impatiens*, which may be shared by other bumble bee species. The use of CO_2_ narcosis to bypass diapause is a well-established technique in bumble bees, and our results demonstrate the flexibility that exists in the timing of administrating CO_2_. Whether CO_2_ narcosis is a viable technique for bypassing diapause in all bumble bee species is unknown and requires additional study; however, in the studied species, it reduced mortality and duration of the solitary phase. Finally, we show that the use of newly emerged workers with and without pupae reduces the time for initiating a colony, in line with findings in other bumble bee species [37,40,57]. These findings, combined with existing data in the literature, can be used to inform and refine future approaches when attempting to rear new species in captivity.

## Data Availability

The datasets generated during and/or analyzed during the current study are available from the corresponding author on reasonable request.
